# HMGB1 inhibition blocks ferroptosis and oxidative stress to ameliorate sepsis‐induced acute lung injury by activating the Nrf2 pathway

**DOI:** 10.1002/kjm2.12851

**Published:** 2024-06-05

**Authors:** Ya‐Jie Jia, Sha Xiong, Ming Yao, Yu Wei, Yan He

**Affiliations:** ^1^ Department of Critical Care Medicine Puren Hospital Affiliated to Wuhan University of Science and Technology Wuhan Hubei China; ^2^ Department of Pharmacy Puren Hospital Affiliated to Wuhan University of Science and Technology Wuhan Hubei China

**Keywords:** acute lung injury, ferroptosis, HMGB1, Nrf2, sepsis

## Abstract

The proinflammatory properties of high‐mobility group box protein 1 (HMGB1) in sepsis have been extensively studied. This study aimed to investigate the impact of HMGB1 on ferroptosis and its molecular mechanism in sepsis‐induced acute lung injury (ALI). A septic mouse model was established using the cecal ligation and puncture method. Blocking HMGB1 resulted in improved survival rates, reduced lung injury, decreased levels of ferroptosis markers (reactive oxygen species, malondialdehyde, and Fe^2+^), and enhanced antioxidant enzyme activities (superoxide dismutase and catalase) in septic mice. In addition, knockdown of HMGB1 reduced cellular permeability, ferroptosis markers, and raised antioxidant enzyme levels in lipopolysaccharide (LPS)‐stimulated MLE‐12 cells. Silencing of HMGB1 led to elevations in the expressions of ferroptosis core‐regulators in LPS‐treated MLE‐12 cells, such as solute carrier family 7 member 11 (SLC7A11), solute carrier family 3 member A2 (SLC3A2), and glutathione peroxidase 4. Furthermore, blocking HMGB1 did not alter ferroptosis, oxidative stress‐related changes, and permeability in LPS‐treated MLE‐12 cells that were pretreated with ferrostatin‐1 (a ferroptosis inhibitor). HMGB1 inhibition also led to elevated expressions of nuclear factor erythroid 2‐related factor 2 (Nrf2) and its downstream targets, heme oxygenase‐1 (HO‐1) and NAD(P)H: quinone oxidoreductase 1 (NQO1) in LPS‐treated MLE‐12 cells and lung tissues from septic mice. The Nrf2‐specific inhibitor ML385 reversed the effects of HMGB1 silencing on ferroptosis and cell permeability in LPS‐treated MLE‐12 cells. Our findings indicated that the inhibition of HMGB1 restrains ferroptosis and oxidative stress, thereby alleviating sepsis‐induced ALI through the activation of Nrf2 signaling.

## 1 INTRODUCTION

Sepsis is a potentially fatal condition that arises due to a dysfunctional host response to infection.^1^ The lung is particularly susceptible to sepsis, as acute lung injury (ALI) is commonly encountered in clinical practice.^2^ ALI triggered by sepsis is characterized by acute inflammation, infiltration of inflammatory cells, edema, and damage to the alveolar epithelium.^3^ Multiple studies have been performed to unravel the intricacies of ALI's pathogenesis, but the underlying mechanisms remain unelucidated. Therefore, gaining a deeper understanding of the molecular mechanisms may promote the development of novel and effective therapeutic strategies for sepsis‐induced ALI.

Oxidative stress‐induced damage plays an essential role in multiple organ failure in sepsis, particularly affecting the lungs.^4^ Patients with sepsis exhibit a decreased antioxidant capacity, which is strongly associated with excessive oxidative stress in the lungs.^5^ In cecal ligation and puncture (CLP)‐induced septic rat models, the lung tissue antioxidant enzyme levels of superoxide dismutase (SOD) and glutathione (GSH) were notably depleted, further emphasizing the significant role of oxidative stress in sepsis‐induced ALI.^6^ Ferroptosis, a newly discovered form of cell death, is characterized by iron‐dependent lipid peroxidation, cell membrane destruction, and extravasation of cell contents, ultimately resulting in cell death.^7^, ^8^ Recent studies have demonstrated that ferroptosis plays a pivotal role in sepsis‐induced multiple organ injury, and its inhibition can alleviate organ damage.^4^, ^9^ Nuclear factor erythroid 2‐related factor 2 (Nrf2) serves as a renowned key transcription mediator for cell adaptation and defense against oxidative stress in various diseases, including cancers and sepsis.^10^ In addition, multiple studies have reported that Nrf2 is involved in ferroptosis‐associated diseases. Wang et al. reported that inhibiting the Nrf2/glutathione peroxidase 4 (GPX4) pathway promotes ferroptosis, thereby exacerbating doxorubicin‐induced cardiomyopathy.^11^ Sun et al. confirmed that activating the Keap1–Nrf2 pathway shields against ferroptosis in hepatocellular carcinoma cells.^12^ These findings suggest that Nrf2 signaling plays a crucial role in regulating ferroptosis and oxidative stress, which may also hold significant implications in septic ALI.

High‐mobility group box 1 protein (HMGB1) is a non‐histone nucleoprotein and plays a pivotal role in inflammatory responses associated with sepsis.^13^ HMGB1 is a reliable predictor of poor prognosis in sepsis patients, and its downregulation has been shown to alleviate ALI in septic mice.^14^ Strategies that aim to inhibit HMGB1 secretion or block its activity through antibody targeting have been shown to provide protection against sepsis‐induced ALI and enhance survival rates in septic animals.^15^ In addition, previous research has established that HMGB1 regulates glucose‐induced ferroptosis through the Nrf2 pathway in mesangial cells.^16^ Zhu et al. further demonstrated that activating the HMGB1/GPX4 pathway and inhibiting ferroptosis effectively mitigated hypoxic–ischemic brain damage in neonatal rats.^17^ Similarly, Du et al. confirmed that the activation of Nrf2 and inhibition of HMGB1 protected melanocytes from oxidative stress.^18^ Despite these findings, the precise mechanism underlying the regulatory effects of HMGB1 on ferroptosis and oxidative stress in sepsis‐induced ALI remains unclear.

In the present study, a sepsis‐induced ALI model was established utilizing mice treated with CLP and murine lung epithelial cells (MLE‐12) exposed to lipopolysaccharide (LPS). Furthermore, the effects of downregulated HMGB1 on ferroptosis and oxidative stress in sepsis‐induced ALI were explored, and the underlying molecular mechanisms were investigated. The findings of this study may provide a novel therapeutic target for ALI.

## 2 MATERIALS AND METHODS

### 2.1 Animal model and delivery of adenoviral vectors

The CLP method was used to establish a sepsis model in mice. Eighty‐four C57BL/6 male mice (6–8 weeks) were purchased from SLAC Lab Animals (Shanghai, China) and raised at a temperature of 22–24°C and a humidity of 60% with a 12 h light/dark cycle. All experiments were approved by the Animal Care and Use Committee of Puren Hospital (Wuhan, China) and followed the Guide for the Care and Use of Laboratory Animals. The mice were randomly assigned to four groups (*n* = 21 in each group, total 84), including the sham group, the sepsis group (model), the adenoviral vectors containing sh‐NC (Ad‐sh‐NC) group, and the adenoviral vectors containing sh‐HMGB1 (Ad‐sh‐HMGB1) group. To establish the animal model of ALI, the mice were anesthetized with pentobarbital sodium via intraperitoneal injection, and an incision (1–2 cm) was performed on the abdomen to expose the cecum. The cecum was ligated using 3‐0 silk sutures at 1 cm from the tip and perforated once with an 18‐gauge needle at 0.5 cm from the ligation. The contents were extruded into the abdominal cavity, and the abdominal musculature and skin were closed. The sham‐operated mice underwent the same surgical procedures without the ligation or puncture of the cecum. Adenoviral vectors containing sh‐HMGB1 (Ad‐sh‐HMGB1) or sh‐NC (Ad‐sh‐NC) were purchased from Genepharma Co., Ltd (Shanghai, China). Subsequently, 1 × 10^8^ virus particles [vp]/20 μL were injected into mice via the tail vein immediately after the CLP operation. After 24 h of CLP, the blood samples of mice (*n* = 12 in each group, total 48) were collected and lung tissues were taken out immediately after euthanasia. Blood samples were centrifuged at 3000 rpm for 20 min at 4°C to obtain the serum. The unanesthetized mice (*n* = 9 in each group, total 36) were subjected to prognostic analysis.

### 2.2 Hematoxylin–eosin staining assay

The lung tissues collected from each group were fixed overnight in 4% formaldehyde. Thereafter, the samples were dehydrated and embedded in paraffin to ensure proper preservation and sectioning. The paraffin‐embedded lung tissue sections were then subjected to hematoxylin–eosin (H&E) staining.

### 2.3 Determination of wet‐to‐dry weight ratio of lung tissue

The filter paper was used to absorb the surface water and blood from the upper lobe of the right lung of each mouse. The wet weight of the lung was then measured using an electronic balance. The lung was then placed in a dryer at 80°C for 24 h to determine the dry weight. Finally, the wet weight to dry weight ratio of the lung was calculated.

### 2.4 Cell culture, transfection, and treatments

The murine lung epithelial cell line (MLE‐12) was obtained from Wuhan Shanen Biotechnology Co., Ltd. (Wuhan, China) and cultured in DMEM/F12 (Gibco, Grand Island, NY, USA) supplemented with 10% fetal bovine serum (Gibco) and 1% penicillin–streptomycin (Millipore, Waltham, MA, USA). The cells were maintained in a controlled environment at 37°C with 5% CO_2_. A total of 1 × 10^6^ MLE‐12 cells were seeded into a 12‐well plate. Cells were then transfected with either 100 nM of HMGB1 siRNA (si‐HMGB1) or control siRNA (si‐NC) using Lipofectamine 2000 reagent (Invitrogen) for a duration of 48 h, following established protocols.^19^ Following transfection, the cells were stimulated by 500 ng/mL LPS (Sigma, St Louis, MO, USA) for 24 h. The si‐HMGB1 and si‐NC were purchased from RiboBio Co., Ltd. (Guangzhou, China). In addition, MLE‐12 cells were incubated with either the ferroptosis inhibitor ferrostatin‐1 (Fer‐1, 2 μM; MedChemExpress) or the Nrf2 inhibitor ML385 (2 μM; MedChemExpress) for 24 h.

### 2.5 Permeability of MLE‐12 cells

After 24 h of LPS stimulation and subsequent phosphate‐buffered saline (PBS) washes, 100 μL of FITC‐dextran (1 μg/mL; MedChemExpress) was placed into the upper chamber, and 600 μL of sterile PBS was added to the lower chamber. This setup was maintained at 37°C for 1 h. Subsequently, the Cytation3 multi‐function detection system (BioTek Instruments, Inc.) was utilized to assess the absorbance value of FITC in the lower chamber. The results were presented as a ratio comparing the experimental group to the control group.

### 2.6 Detection of antioxidant enzymes

The lung tissues were homogenized and centrifuged, and the supernatants were collected. Moreover, the MLE‐12 cells were lysed, and their supernatants were gathered. The activities of SOD and catalase (CAT) as well as the content of malondialdehyde (MDA) were measured in both mouse lung tissues and MLE‐12 cells using enzyme‐linked immunosorbent assay (ELISA) kits obtained from Jiancheng Bioengineering Institute (Nanjing, China). All measurements were performed according to the manufacturer's instructions.

### 2.7 Detection of reactive oxygen species level in MLE‐12 cells

The reactive oxygen species (ROS) levels in MLE‐12 cells were assayed through DCFH‐DA staining. Cells were incubated with 5 μM DCFH‐DA (Beyotime) at 37°C for 20 min in a dark environment. Then, the samples were washed three times with PBS to remove unbound dyes. Finally, the DCF fluorescence was measured using CytoFlex S Flow cytometry (Beckman Coulter, CA, USA).

### 2.8 Measurement of ROS production in lung tissue

Approximately 100 mg of lung tissues were homogenized in PBS and a single‐cell suspension was prepared using enzymatic digestion.^20^ ROS production was then assayed using a commercial ROS Fluorometric Assay Kit (Elabscience, Wuhan, China), following the manufacturer's guidelines. Fluorescence intensity was determined using a fluorescence microplate reader, with an excitation wavelength of 488 nm and an emission wavelength of 525 nm.

### 2.9 Determination of Fe^2+^ level

The Iron Assay Kit (Abcam, UK) was employed to determine Fe^2+^ levels in lung tissues and MLE‐12 cells, according to the manufacturer's instructions. Initially, lung tissues were homogenized and centrifuged, and the supernatants were collected. Similarly, MLE‐12 cells were lysed to obtain supernatants. Next, 100 μL of supernatant was mixed with 5 μL of iron buffer and incubated at 37°C for 30 min. Immediately following incubation, the optical density value was measured at 593 nm.

### 2.10 Enzyme‐linked immunosorbent assay

The levels of interleukin‐1β (IL‐1β) and IL‐18 in serum and MLE‐12 cell supernatants were quantified using ELISA kits specific for each cytokine (R&D, USA). Similarly, the HMGB1 levels in both serum and MLE‐12 cell supernatants were assayed using a mouse HMGB‐1 ELISA Kit (Elabscience, Wuhan, China). The absorbance at 450 nm was recorded using a microplate reader (Bio‐Rad, Hercules, CA).

### 2.11 Western blot assay

Total proteins were extracted from homogenized mouse lung tissues and MLE‐12 cells using RIPA buffer (Beyotime) and were quantified using a BCA kit (Beyotime). Subsequently, the protein samples were denatured, separated on a 10% SDS‐PAGE gel, and transferred onto PVDF membranes (Millipore, Burlington, USA). The membranes were blocked with 5% non‐fat milk for 1 h at room temperature, followed by overnight incubation at 4°C with primary antibodies specific for Nrf2 (ab137550, 1:1000; Abcam, UK), HMGB1 (ab18256, 1:1000; Abcam), HO‐1 (ab189491, 1:1000; Abcam), SLC7A11 (1:1000, Beyotime), SLC3A2 (abs136980, 1:1000; Absin, China), GPX4 (ab125066, 1:1000; Abcam), NQO1 (ab80588, 1:1000; Abcam), and GAPDH (ab8245, 1:1000; Abcam). After washing with TBST three times, the membranes were incubated with the corresponding goat anti‐rabbit secondary antibodies (ab6721, 1:5000; Abcam) for 1 h at room temperature. Finally, the membranes were treated with an enhanced chemiluminescence (ECL) substrate, and images were captured using a gel imaging scanning system (Bio‐Rad, USA).

### 2.12 Statistical analysis

Data were presented as mean ± standard deviation. Statistical analysis was conducted using GraphPad Prism 7.0 software (GraphPad Software Inc., CA, USA). Differences among various groups were analyzed using one‐way ANOVA, followed by Tukey's post hoc test. The Kaplan–Meier method with log rank testing was employed for survival data analysis. A *p* < 0.05 was considered statistically significant.

## 3 RESULTS

### 3.1 Downregulation of HMGB1 mitigates ferroptosis and oxidative stress in septic mice

**FIGURE 1:**
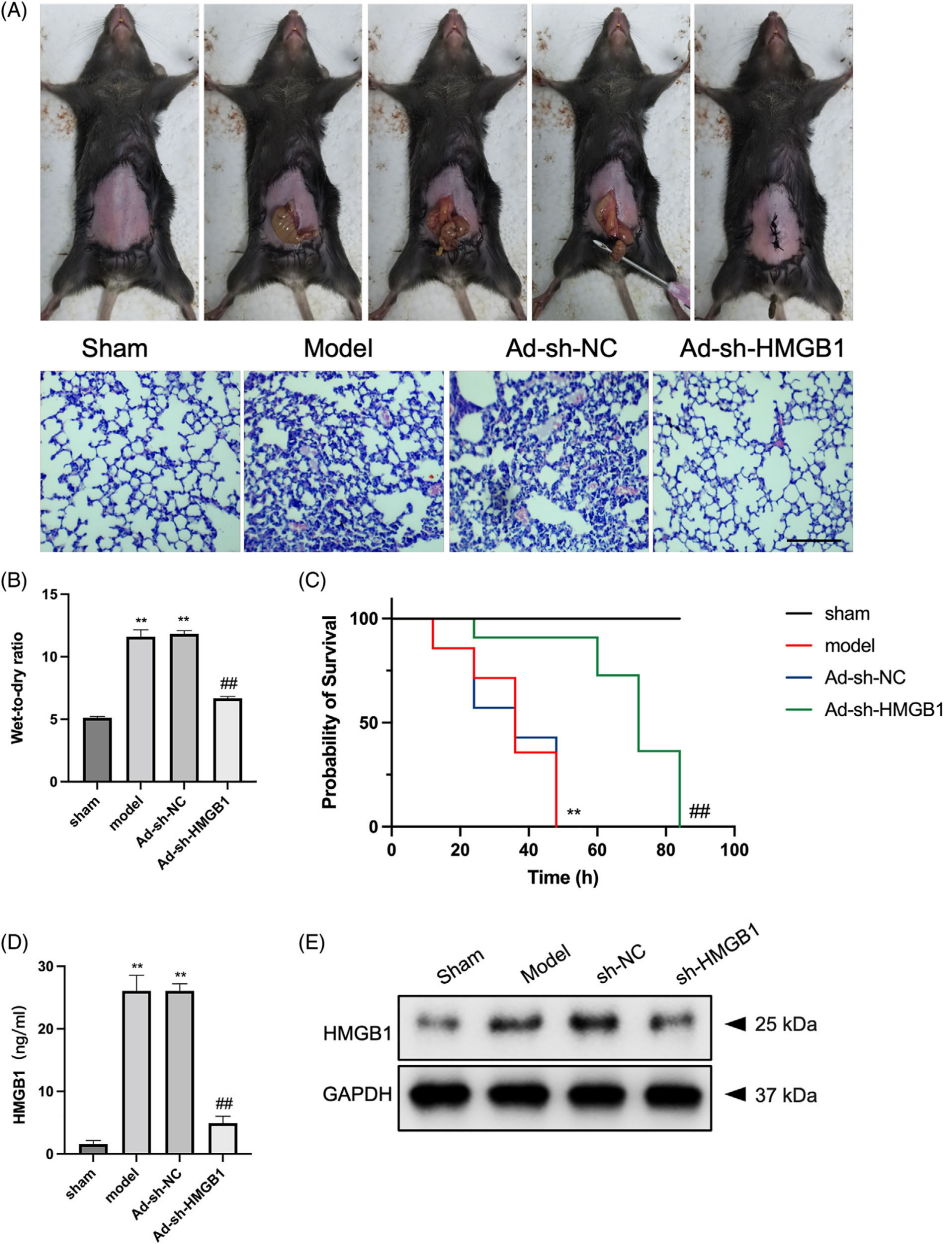
Downregulated HMGB1 alleviates ALI in C57BL/6 mice with sepsis. (A) Mice were injected with Ad‐sh‐NC or Ad‐sh‐HMGB1 through the tail vein immediately after the CLP operation. Schematic diagram of the construction of sepsis in C57BL/6 mice (upper panel) (*n* = 12). H&E staining of lung tissues from each group of ALI mice (lower panel). (B) Ad‐sh‐HMGB1 reduced the lung wet‐to‐dry ratio in mice with CLP‐induced ALI. (C) Ad‐sh‐HMGB1 improved survival in mice with ALI from sepsis (*n* = 9). (D, E) HMGB1 levels in serum and lung tissues of mice were measured by ELISA and Western blot assays. **p < 0.01 versus sham; ^##^ *p* < 0.01 versus model.

An ALI model was established in C57BL/6 mice through CLP surgery to explore the impact of HMGB1 downregulation on ferroptosis and oxidative stress in sepsis‐induced ALI. H&E staining was performed to assess the changes in pathological lung tissues. The lung tissue samples from the sham group exhibited a clear structure, with no edematous zones or inflammation. Conversely, pronounced inflammatory cell infiltration and edema were observed in the lungs of the sepsis (model) group. Notably, the lung tissue morphology in the Ad‐sh‐HMGB1‐treated group resembled that of the sham group (Figure 1A). The wet/dry ratio, a marker of lung edema, was significantly elevated in sepsis mice but was effectively reduced by Ad‐sh‐HMGB1 administration (Figure 1B). Furthermore, Ad‐sh‐HMGB1 treatment significantly improved the survival rate in septic ALI mice (Figure 1C). Injection of Ad‐sh‐HMGB1 in CLP‐treated mice resulted in reduced HMGB1 levels in both serum and lung tissues (Figure 1D,E). These findings suggest that HMGB1 downregulation may attenuate sepsis‐induced ALI.

**FIGURE 2:**
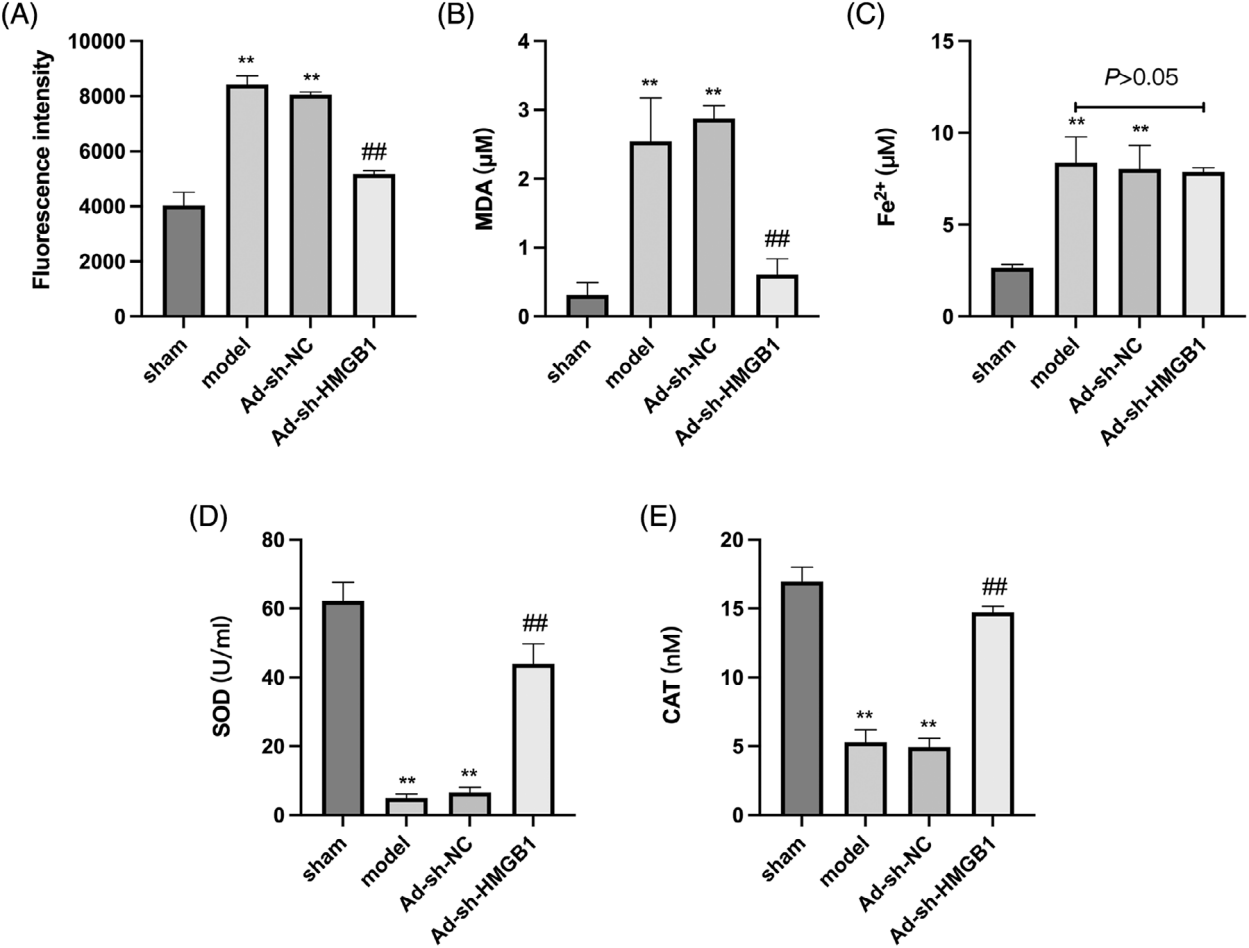
Downregulated HMGB1 abates ferroptosis and oxidative stress in septic mice. (A) The level of ROS in the lung from septic mice was detected using a ROS fluorometric assay kit. (B, C) MDA and Fe^2+^ levels in the lung tissues of mice were detected by corresponding kits. (D, E) SOD and CAT levels in the lung tissues of mice were detected by ELISA. **p < 0.01 versus sham; ^##^ *p* < 0.01 versus model.

In addition, the model group demonstrated elevated levels of ferroptosis markers (ROS, MDA, and Fe^2+^) and reduced levels of antioxidant stress markers (SOD and CAT) in lung tissues compared to the sham group. However, the downregulation of HMGB1 attenuated the increase in ROS and MDA levels induced by CLP, without affecting Fe^2+^ levels (Figure 2A–C). Concurrently, HMGB1 downregulation restored SOD and CAT levels in lung tissues of septic mice induced by CLP (Figure 2D,E). Collectively, these findings suggested that the downregulation of HMGB1 exerts protective effects against sepsis‐induced lung injury, mitigating ferroptosis and oxidative stress during the CLP‐induced lung injury process.

### 3.2 Silencing of HMGB1 attenuates ferroptosis and oxidative stress in LPS‐stimulated MLE‐12 cells

**FIGURE 3:**
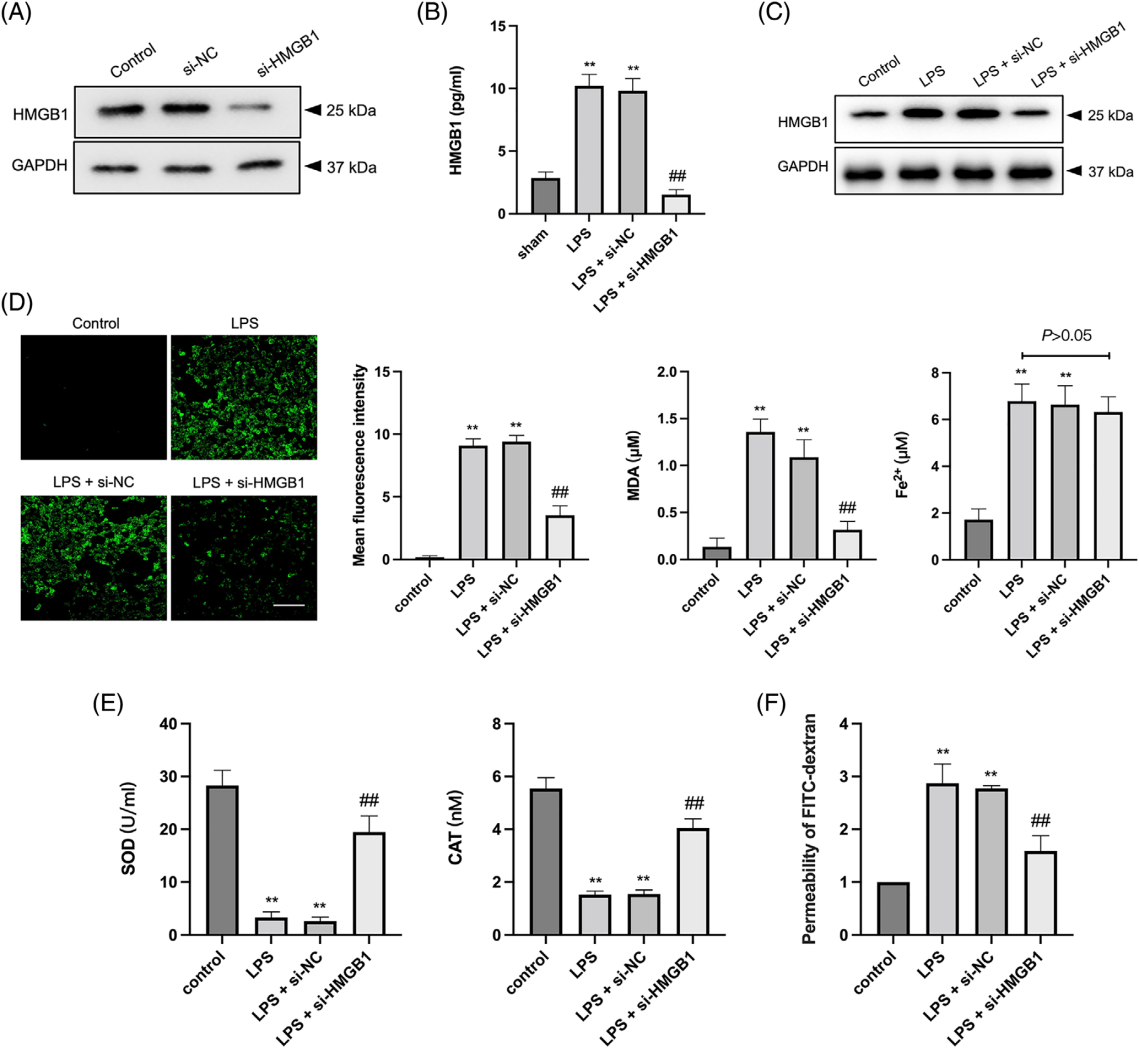
HMGB1 silencing inhibits ferroptosis and oxidative stress in LPS‐treated MLE‐12 cells. (A) The si‐HMGB1 was transfected into MLE‐12 cells and the expression of HMGB1 was determined by western blot. (B, C) After transfected with si‐HMGB1, MLE‐12 cells were stimulated by LPS for 24 h. ELISA and Western blot were used to measure HMGB1 protein levels in supernatants and cells. (D) ROS, MDA, and Fe^2+^ in LPS‐stimulated MLE‐12 cells were assessed by corresponding kits. (E) SOD and CAT levels in LPS‐stimulated MLE‐12 cells were assessed by corresponding kits. (F) The permeability of MLE‐12 cells was measured by FITC‐dextran absorbance value. **p < 0.01 versus control; ^##^ *p* < 0.01 versus LPS.

Furthermore, an LPS‐stimulated injury model was constructed using MLE‐12 cells to investigate the impact of HMGB1 silencing on ferroptosis and oxidative stress. siRNA‐mediated knockdown of HMGB1 (si‐HMGB1) was performed to block HMGB1 expression in these cells (Figure 3A). Both ELISA and Western blot analysis revealed that LPS stimulation elevated HMGB1 levels in both cell supernatants and lysates, which was effectively abrogated by HMGB1 silencing (Figure 3B,C). In addition, increased levels of ferroptosis markers (ROS, MDA, and Fe^2+^) and decreased levels of antioxidant enzymes (SOD and CAT) were observed in LPS‐treated MLE‐12 cells. However, si‐HMGB1 transfection reversed the LPS‐induced changes in protein levels, except for Fe^2+^ (Figure 3D,E). Moreover, LPS stimulation resulted in increased permeability of MLE‐12 cells, which was attenuated by HMGB1 silencing (Figure 3F). Collectively, these findings demonstrated that silencing HMGB1 exerts inhibitory effects on ferroptosis and oxidative stress in LPS‐treated pulmonary epithelial cells, providing further evidence for the protective role of HMGB1 downregulation in lung injury.

### 3.3 Blocking HMGB1 mitigates LPS‐induced injury in MLE‐12 cells by suppressing ferroptosis

**FIGURE 4:**
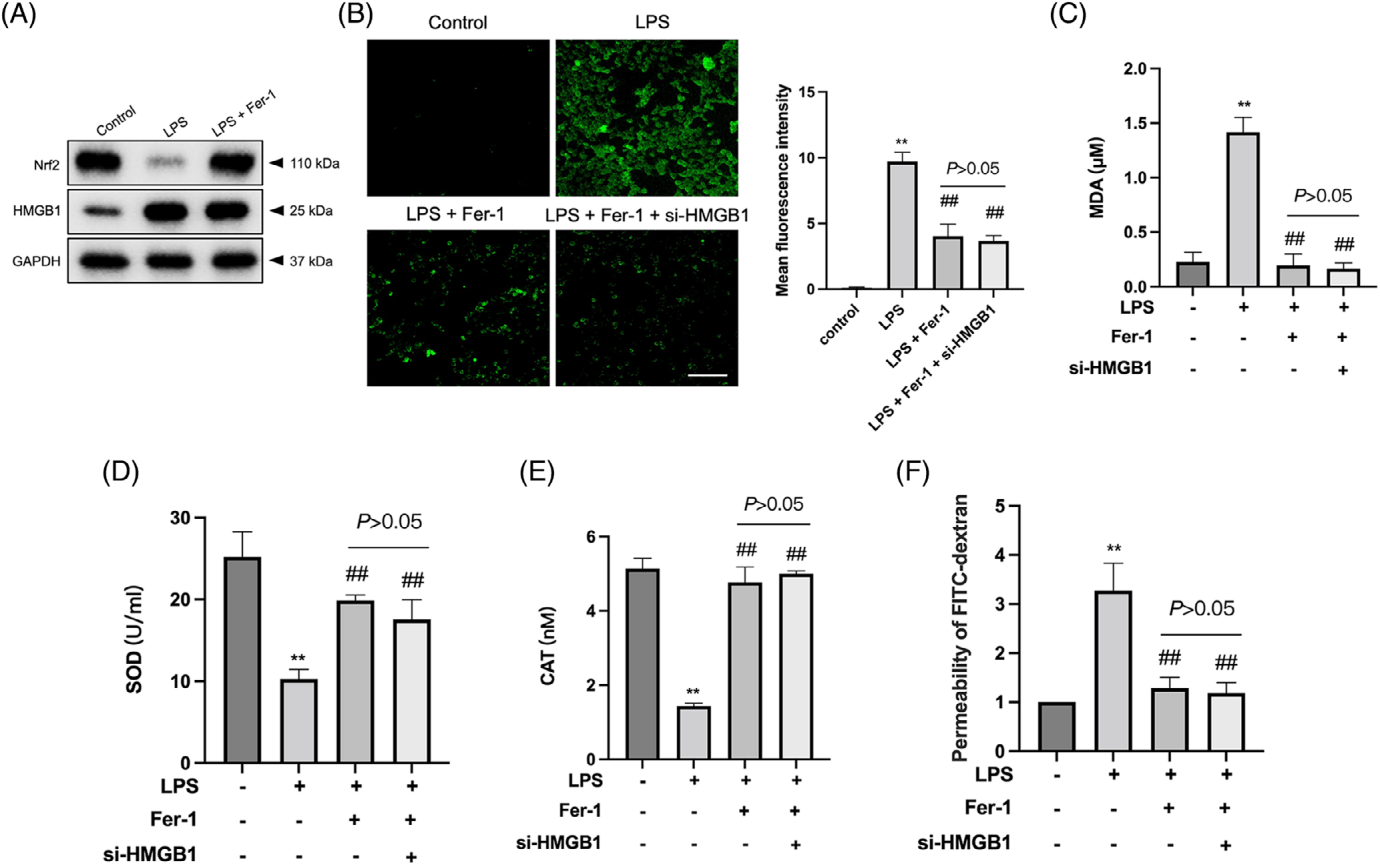
Blocking HMGB1 represses LPS‐induced MLE‐12 injury via inhibition of ferroptosis. (A) The expressions of HMGB1 and Nrf2 in 2 μM Fer‐1 treated MLE‐12 cells were detected by Western blot. (B, C) In the presence of LPS (500 ng/mL), MLE‐12 cells were treated with 2 μM Fer‐1 or Fer‐1 combination with si‐HMGB1. ROS and MDA levels in LPS‐stimulated MLE‐12 cells were detected by corresponding kits. (D, E) SOD and CAT levels in LPS‐stimulated MLE‐12 cells were detected by corresponding kits. (F) The permeability of the MLE‐12 was assessed. **p < 0.01 versus control group; ^##^ *p* < 0.01 versus LPS group.

To further investigate whether the attenuation of LPS‐induced MLE‐12 cell injury by HMGB1 silencing is dependent on ferroptosis, LPS‐stimulated cells were treated with 2 μM ferrostatin‐1 (Fer‐1), a specific ferroptosis inhibitor.^21^ As demonstrated in Figure 4A, after Fer‐1 administration, the expression of Nrf2 protein was upregulated in LPS‐treated MLE‐1 cells, whereas the expression of HMGB1 protein remained unchanged.^22^ Fer‐1 also effectively suppressed the accumulation of ROS and MDA, while the levels of antioxidant enzymes such as SOD and CAT were increased in LPS‐treated MLE‐12 cells (Figure 4B–E). Notably, in the presence of Fer‐1, HMGB1 did not exert any additional inhibitory effect on ferroptosis in LPS‐treated MLE‐12 cells. Furthermore, the addition of Fer‐1 reduced the permeability of LPS‐treated MLE‐12 cells. However, the presence of si‐HMGB1 did not affect the reduction in permeability caused by the Fer‐1 treatment (Figure 4F). These observations suggested that HMGB1 silencing protects against LPS‐induced injury in MLE‐12 cells by suppressing ferroptosis, providing additional mechanistic insights into the role of HMGB1 in pulmonary epithelial cell damage.

### 3.4 Interference of HMGB1 represses ferroptosis in LPS‐treated MLE‐12 cells by regulating the Nrf2 pathway

**FIGURE 5:**
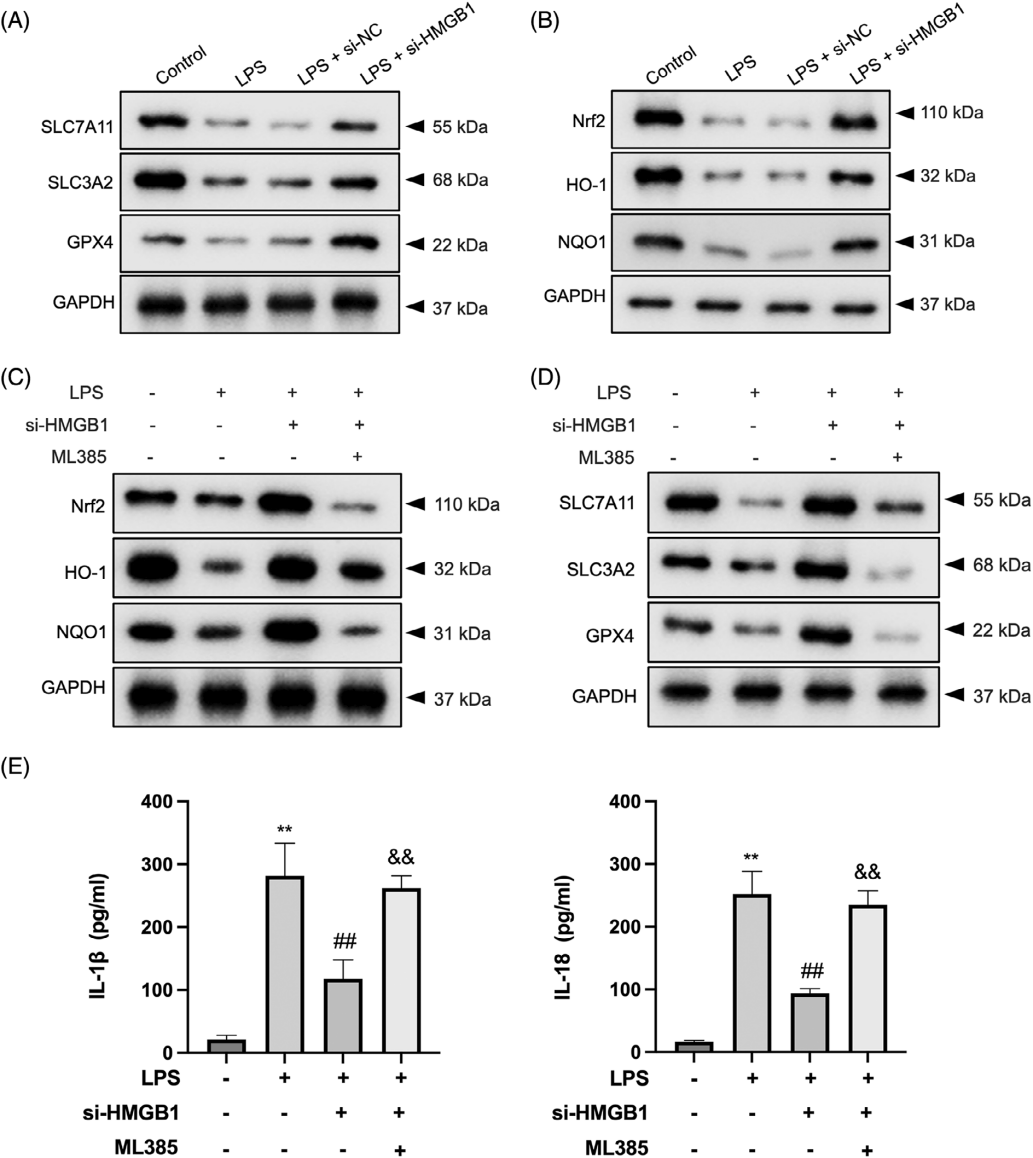
Interference of HMGB1 represses ferroptosis in LPS‐treated MLE‐12 cells via regulating the Nrf2 pathway. (A) After transfected with si‐HMGB1, MLE‐12 cells were stimulated by LPS for 24 h. Western blot was employed to measure the protein levels of SLC7A11, SLC3A2, and GPX4 in MLE‐12 cells. (B) The expressions of Nrf2, HO‐1, and NQO1 in MLE‐12 cells were detected by Western blot. (C) Cells were treated with si‐HMGB1 or si‐HMGB1 in combination with 2 μM ML385. Western blot was used to measure the protein levels of Nrf2, HO‐1, and NQO1. (D) Cells were treated with si‐HMGB1 or si‐HMGB1 in combination with 2 μM ML385. Western blot was used to measure the protein levels of SLC7A11, SLC3A2, and GPX4. (E) IL‐1β and IL‐18 levels in LPS‐stimulated MLE‐12 cells supernatants were assessed by the ELISA method. **p < 0.01 versus control group; ^##^ *p* < 0.01 versus LPS group; ^&&^ *p* < 0.01 versus LPS + si‐HMGB1 group.

Ferroptosis is triggered through two primary mechanisms: the enzyme‐regulated or intrinsic pathway, which involves the inhibition of system xc−, and the transporter‐dependent or extrinsic pathway, which directly targets GPX4 and induces lipid peroxidation.^23^ The cystine/glutamic acid exchanger, known as system xc−, comprises two subunits, namely the light chain subunit SLC7A11 and the heavy chain subunit SLC3A2. Together, they maintain GSH production, a crucial endogenous antioxidant, by facilitating the exchange of extracellular cystine for intracellular glutamate.^24^ Upon silencing of HMGB1, a significant elevation in the reduced expressions of SLC7A11 and SLC3A2 was observed in LPS‐treated MLE‐12 cells (Figure 5A). In the intrinsic pathway, GSH plays a pivotal role as the major antioxidant in cells. GPX4 utilizes reduced GSH to scavenge lipid hydroperoxides, thereby safeguarding cells from ferroptosis. On the other hand, the inactivation of GPX4 leads to the accumulation of lipid peroxides and triggers ferroptosis. Interestingly, HMGB1 silencing was found to enhance the protein level of GPX4 in LPS‐treated MLE‐12 cells (Figure 5A).

Nrf2 is a pivotal transcription factor that plays a crucial role in regulating oxidative stress and maintaining intracellular redox homeostasis.^25^ Nrf2 has been shown to exert its anti‐ferroptosis function by modulating the expression of GPX4 and SLC7A11.^11^, ^26^ Therefore, experiments were conducted to determine whether HMGB1 silencing affects ferroptosis through the Nrf2 signaling axis in LPS‐treated MLE‐12 cells. The LPS‐treated MLE‐12 cells demonstrated downregulation of Nrf2 and its target proteins, HO‐1 and NQO1. However, the expressions of Nrf2, HO‐1, and NQO1 were significantly upregulated upon si‐HMGB1 transfection (Figure 5B). Administration of the Nrf2 inhibitor ML385 (2 μM) effectively abrogated the upregulation of Nrf2, HO‐1, and NQO1 caused by si‐HMGB1 transfection (Figure 5C). Moreover, administration of the Nrf2 inhibitor ML385 inhibited the upregulation of SLC7A11, SLC3A2, and GPX4 caused by si‐HMGB1 transfection (Figure 5D). Furthermore, si‐HMGB1 reduced ferroptosis markers (ROS and MDA), elevated antioxidant enzyme levels (SOD and CAT), and decreased cell permeability in LPS‐treated cells (Figure S1A–E). In contrast, ML385 reversed these effects, leading to an increase in ferroptosis markers, cell permeability, and a decrease in antioxidant enzyme levels in MLE‐12 cells (Figure S1A–E). si‐HMGB1 also significantly reduced the levels of IL‐1β and IL‐18 in the supernatants of LPS‐treated MLE‐12 cells, while ML385 restored the levels of these inflammatory markers (Figure 5E). Collectively, these findings indicated that knockdown of HMGB1 inhibits ferroptosis in LPS‐treated MLE‐12 cells by activating the Nrf2 signaling pathway.

### 3.5 Deletion of HMGB1 activates Nrf2 signaling in septic mice

**FIGURE 6:**
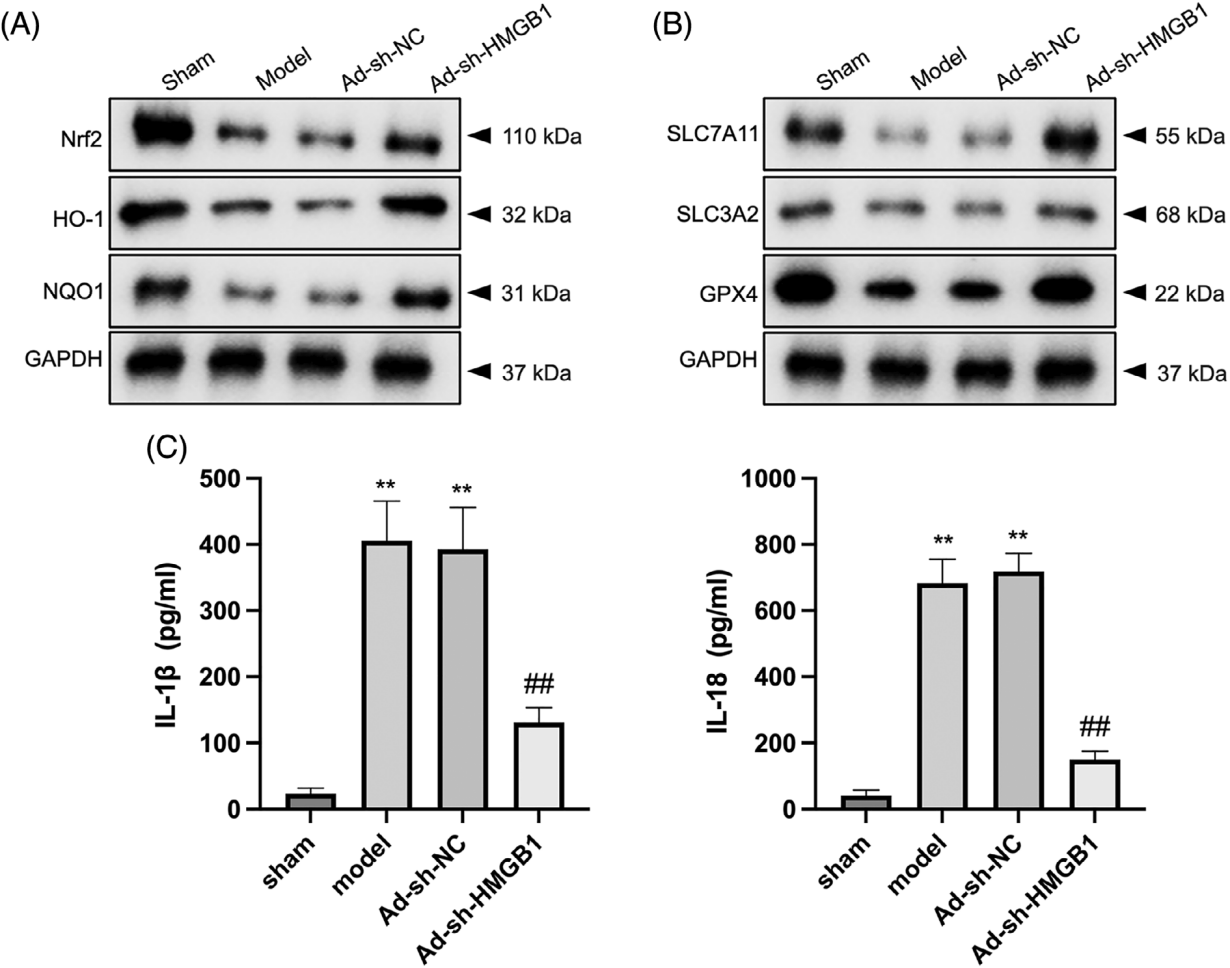
Deletion of HMGB1 activates Nrf2 signaling in septic mice. Mice were injected with Ad‐sh‐HMGB1 or Ad‐sh‐NC via the tail vein immediately after CLP surgery. (A) Western blot was carried out to measure the protein levels of Nrf2, HO‐1, and NQO1 in the lung tissues of mice. (B) Measurement of the protein levels of SLC7A11, SLC3A2, and GPX4 in the lung tissues of mice. (C) Serum IL‐1β and IL‐18 levels of mice were determined by ELISA. **p < 0.01 versus sham; ^##^ *p* < 0.01 versus model.

The role of HMGB1 downregulation in mitigating ferroptosis and oxidative stress by potentiating Nrf2 signaling was confirmed in vitro, so this mechanism was further explored in vivo. As demonstrated in Figure 6A, mice subjected to CLP surgery exhibited significant suppression of Nrf2 signaling, as evidenced by reduced protein expressions of Nrf2, HO‐1, and NQO1 in lung tissues. However, the introduction of Ad‐sh‐HMGB1 reversed these alterations, indicating that HMGB1 deletion activates Nrf2 signaling. Similarly, CLP‐induced mice demonstrated reduced expressions of SLC7A11, SLC3A2, and GPX4, while the Ad‐sh‐HMGB1 injection abrogated this inhibition (Figure 6B). In addition, serum levels of inflammatory markers IL‐1β and IL‐18 were elevated in CLP‐induced mice compared to sham‐operated controls. Notably, HMGB1 inhibition significantly reduced these levels (Figure 6C), further supporting the anti‐inflammatory role of HMGB1 deletion. Taken together, these findings highlight the role of HMGB1 deletion in activating Nrf2 signaling and mitigating inflammation in a murine model of sepsis.

## 4 DISCUSSION

During the progression of sepsis, the elevated ferroptosis and oxidative stress within the alveoli play a pivotal role in the formation of pulmonary edema and subsequent lung tissue damage.^4^, ^27^ Patients diagnosed with sepsis‐induced ALI exhibit a significantly higher rate of organ dysfunction and mortality compared to those with non‐sepsis‐induced ALI.^28^ The present study established a murine model of sepsis and demonstrated that HMGB1 blockade effectively mitigates ferroptosis and oxidative stress while concurrently activating the Nrf2 signaling pathway. Treatment with Fer‐1, a ferroptosis inhibitor, was found to suppress ferroptosis and oxidative stress. However, upon treated with Fer‐1, these effects were not influenced by HMGB1 blockade in the LPS‐stimulated MLE‐12 injury model. Furthermore, the Nrf2 inhibitor ML385 abrogated the protective effects of HMGB1 blockade on ferroptosis and oxidative stress observed in vitro. These findings underscore the crucial role of the Nrf2 signaling pathway in regulating ferroptosis and oxidative stress in sepsis‐induced ALI following HMGB1 blockade.

Oxidative stress is defined as a disruption in the delicate balance between ROS production and antioxidant defense mechanisms and has been implicated as an essential driver in the progression of sepsis‐induced ALI.^29^ Accumulating evidence indicates that ferroptosis, a distinct form of cell death associated with ALI in sepsis, alters cell morphology and gives rise to smaller mitochondria, distinguishing it from apoptosis and necrosis.^30^ A crucial aspect of ferroptosis is the iron‐dependent oxidation of lipids, leading to excessive ROS generation.^31^ CLP‐treated C57BL/6 mice and LPS‐stimulated MLE‐12 cells have been widely utilized to establish models of sepsis‐induced ALI.^32^ The current study revealed that the downregulation of HMGB1 reduced the levels of ferroptosis markers ROS and MDA without affecting Fe^2+^ accumulation. In addition, HMGB1 blockade elevated the levels of antioxidants such as SOD and CAT in the lung tissues of CLP‐treated mice and LPS‐stimulated MLE‐12 cells. These findings suggested that blocking HMGB1 suppresses ferroptosis and oxidative stress in sepsis, highlighting the potential therapeutic implications of targeting this pathway.

Fer‐1 is a ferroptosis inhibitor that effectively fortifies antioxidant defenses. In LPS‐treated MLE‐12 cells, Fer‐1 treatment effectively limited the levels of ferroptosis markers ROS and MDA, while increasing the levels of antioxidant enzymes such as SOD and CAT. Notably, Fer‐1‐induced changes in oxidative stress and ferroptosis were not influenced by HMGB1 silencing. In addition, Fer‐1 treatment reduced cell permeability under LPS stimulation, indicating its protective effects. These findings revealed that blocking HMGB1 suppresses LPS‐induced MLE‐12 injury by inhibiting ferroptosis. Nrf2 is a crucial regulator of antioxidant defense and plays a central role in maintaining redox homeostasis by regulating its downstream target genes.^33^ Previous studies have reported that Nrf2 activation restricts ferroptosis by upregulating genes involved in iron and ROS metabolism.^26^ Wu et al. demonstrated that HMGB1 knockdown suppresses ferroptosis and enhances Nrf2 expression and its downstream targets in mesangial cells exposed to high glucose conditions.^16^ Similarly, Wang et al. showed that aucubin mitigates oxidative stress and inflammation by triggering Nrf2‐induced antioxidant activities in mice with traumatic brain injury.^34^ This study further observed that HMGB1 deletion activated Nrf2 signaling in the lung tissues of CLP‐treated mice and LPS‐stimulated MLE‐12 cells. Therefore, HMGB1 inhibition may attenuate LPS‐induced ALI by activating Nrf2‐mediated antioxidant defense mechanisms.

Ultimately, experiments were carried out to determine whether silencing of HMGB1 can influence ferroptosis via Nrf2 signaling under LPS exposure. Ferroptosis is initiated through both the intrinsic or enzyme‐regulated pathway and the extrinsic or transporter‐dependent pathway. Therefore, the impact of HMGB1 on the expressions of system xc− (composed of SLC7A11 and SLC3A2) and GPX4 was evaluated. In LPS‐treated MLE‐12 cells, HMGB1 silencing increased the expression levels of SLC7A11, SLC3A2, and GPX4, whereas the Nrf2 inhibitor ML385 reversed these changes. These findings indicated that HMGB1 silencing inhibits ferroptosis by enhancing Nrf2 signaling. Our in vivo experiments demonstrated that HMGB1 deletion activated Nrf2 signaling in CLP‐treated mice. Consistent with our findings, Qiu et al. reported suppressed ferroptosis in a mouse model of sepsis‐induced acute kidney injury following activation of the Nrf2/HO‐1 signaling.^35^ Previous studies have shown that Nrf2 induction can inhibit HMGB1 expression in septic diseases. For instance, Yang et al. demonstrated that activation of the Nrf2/HO‐1 signaling could reduce HMGB1‐mediated septic response and improve the survival of septic mice.^36^ In addition, in certain inflammatory disorders, including traumatic brain injury, cerebral ischemia–reperfusion injury, and diabetic kidney disease, HMGB1 exhibits a negative regulatory role toward Nrf2.^16^, ^37^ In glucose‐treated mesangial cells, HMGB1 knockdown promotes Nrf2 expression as well as its downstream targets, including HO‐1 and NQO‐1.^16^ Furthermore, HMGB1 significantly ameliorates lung injury induced by ischemia–reperfusion and alleviates alveolar macrophage pyroptosis by activating the Nrf2/HO‐1 pathway.^38^ The aforementioned studies consistently indicate that HMGB1 exerts a negative feedback regulatory effect on Nrf2. Reducing the level of ferroptosis and inflammatory response can attenuate the lung epithelial cell injury caused by sepsis.^39^ Our study first demonstrated that suppressing HMGB1 can upregulate the Nrf2 pathway to alleviate sepsis‐induced ALI by inhibiting ferroptosis and inflammation.

This study has certain limitations. First, we have not yet specifically investigated the mechanism underlying how HMGB1 regulates Nrf2. Second, we have not validated the impact of HMGB1 on lung injury in a mouse model by targeting the Nrf2 signaling pathway using an Nrf2 inhibitor. Third, the absence of clinical samples limited our ability to confirm the association between HMGB1 and Nrf2, as well as markers of ferroptosis and oxidative stress. The suppressive effect of si‐HMGB11 on ferroptosis is the key experimental conclusion of our study. To further investigate the potential inhibitory effect of siHMGB1 on pyroptosis, we plan to utilize a pyroptosis agonist, Polyphyllin VI, which functions by activating the nucleotide‐binding and oligomerisation domain‐like receptor (NLR) family pyrin domain (PYD)‐containing 3 (NLRP3).^40^ Pyroptosis activation encompasses both the caspase‐1‐dependent classical pathway and the caspase‐1‐independent non‐classical pathway. In our subsequent experiments, we intend to explore the individual effects of si‐HMGB11 on these two distinct mechanisms.

**FIGURE 7:**
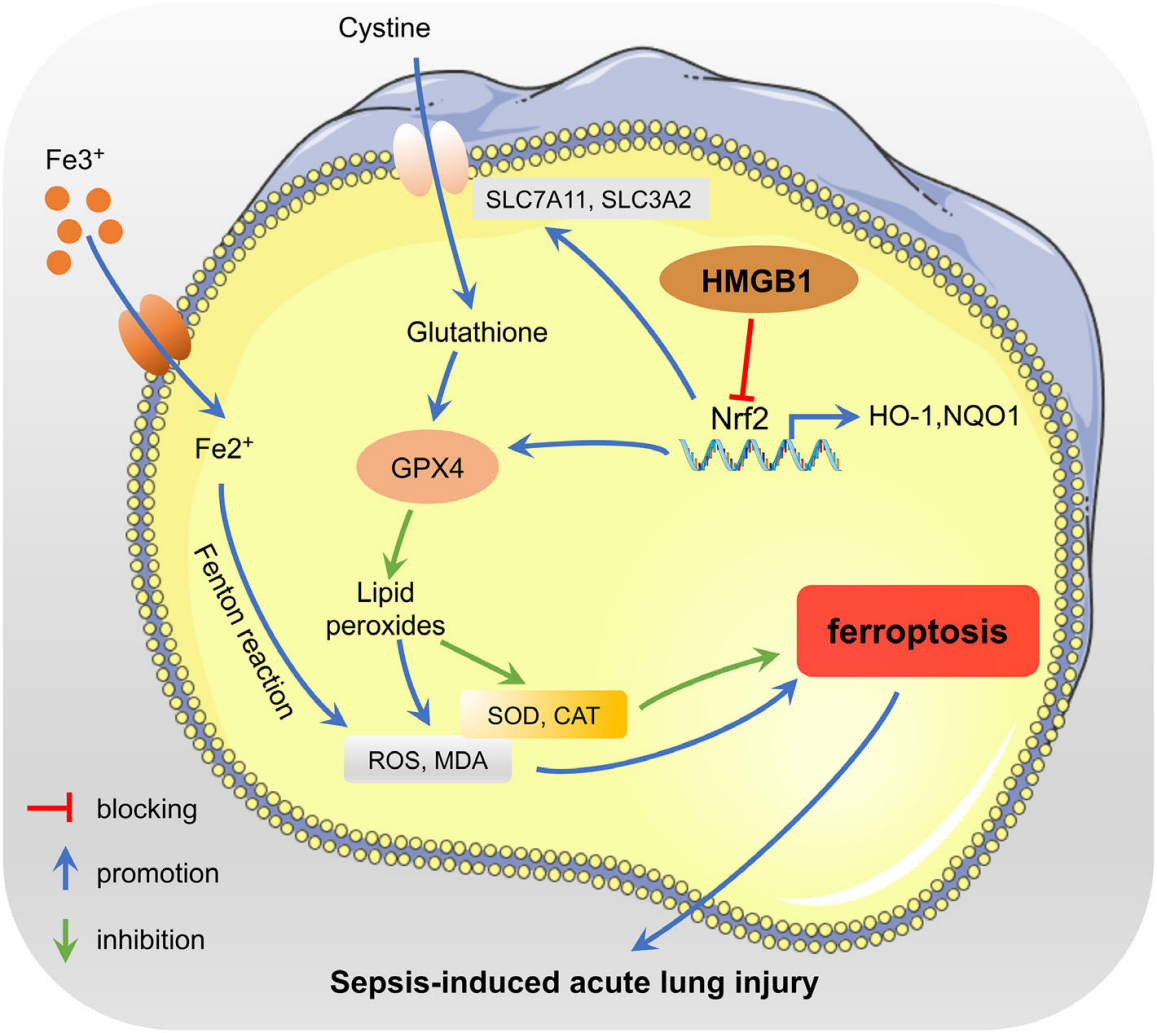
Graphical abstract for HMGB1 leads to ferroptosis in sepsis‐induced ALI. In sepsis‐induced ALI, abnormally elevated HMGB1 inactivates the Nrf2 signaling pathway and further promotes oxidative stress and ferroptosis. Inhibition of HMGB1 activates the Nrf2 pathway, raises SLC7A11, SLC3A2, and GPX4 expression, and alleviates oxidative stress to ameliorate sepsis‐induced ALI.

In summary, the present study revealed that inhibiting HMGB1 effectively mitigates ferroptosis and oxidative stress in sepsis‐induced ALI by activating Nrf2 signaling (Figure 7). This study offers novel insights into the pathogenesis of ALI induced by sepsis and provides a scientific basis for enhanced understanding and potential therapeutic interventions.

## CONFLICT OF INTEREST STATEMENT

The authors declare no conflict of interest.

## Supporting information


**Figure S1.** Blocking HMGB1 represses ferroptosis and cell permeability in LPS‐treated MLE‐12 cells. After treated with si‐HMGB1 or si‐HMGB1 combination with 2 μM ML385. (A, B) ROS and MDA levels in LPS‐stimulated MLE‐12 cells were detected by corresponding kits. (C, D) SOD and CAT levels in LPS‐stimulated MLE‐12 cells were detected by ELISA method. (E) The permeability of the MLE‐12 cells was assessed. **p < 0.01 vs. control cells; ^##^ *p* < 0.01 vs. LPS group; ^&&^ *p* < 0.01 vs. LPS + si‐HMGB1 group.
