# Supplementary material for: HMGB1 inhibition blocks ferroptosis and oxidative stress to ameliorate sepsis‐induced acute lung injury by activating the Nrf2 pathway
Source: Kaohsiung J Med Sci. 2024 Jun 5;40(8):710–21. doi: 10.1002/kjm2.12851 (PMC11895617; doi:10.1002/kjm2.12851)
Supplement: Supplementary file 1 — Figure S1. Blocking HMGB1 represses ferroptosis and cell permeability in LPS‐treated MLE‐12 cells. After treated with si‐HMGB1 or si‐HMGB1 combination with 2 μM ML385. (A, B) ROS and MDA levels in LPS‐stimulated MLE‐12 cells were detected by corresponding kits. (C, D) SOD and CAT levels in LPS‐stimulated MLE‐12 cells were detected by ELISA method. (E) The permeability of the MLE‐12 cells was assessed. **p < 0.01 vs. control cells; ## p < 0.01 vs. LPS group; && p < 0.01 vs. LPS + si‐HMGB1 group. [file KJM2-40-710-s001.docx]

**
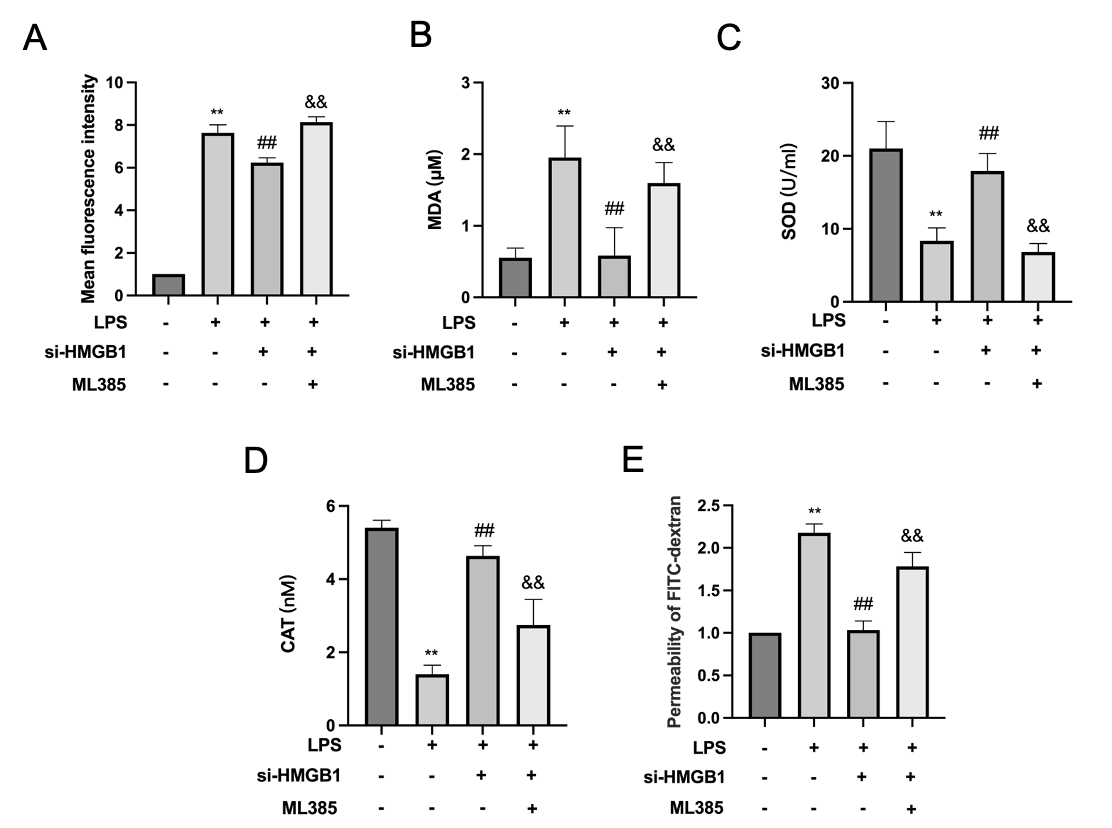
**

**Supplementary Figure 1.** **Blocking HMGB1 represses ferroptosis and cell permeability in LPS-treated MLE-12 cells.** After treated with si-HMGB1 or si-HMGB1 combination with 2 µM ML385. (A-B) ROS and MDA levels in LPS-stimulated MLE-12 cells were detected by corresponding kits. (C-D) SOD and CAT levels in LPS-stimulated MLE-12 cells were detected by ELISA method. (E) The permeability of the MLE-12 cells was assessed. ^**^*P*<0.01 *vs*. control cells; ^##^*P*<0.01 *vs*. LPS group; ^&&^*P*<0.01 *vs*. LPS + si-HMGB1 group.
